# Diagnosis and Treatment of Adrenal Medullary Hyperplasia: Experience from 12 Cases

**DOI:** 10.1155/2014/752410

**Published:** 2014-08-27

**Authors:** Lu Yang, Liang Gao, Xiao Lv, Shengqiang Qian, Siyuan Bu, Qiang Wei, Jiuhong Yuan, Tianyong Fan

**Affiliations:** ^1^Department of Urology, West China Hospital, Sichuan University, No. 37 Guoxue Xiang, Chengdu 610041, China; ^2^Department of Urology, People's Hospital of Deyang City, Deyang 618000, China

## Abstract

*Objective*. To dissect the characteristics of adrenal medullary hyperplasia (AMH) and share our experience of diagnosis and treatment of AMH. *Methods*. From 1999 to 2013, 12 cases of AMH have been pathologically diagnosed after operation in our hospital. The clinical characteristics, process of diagnosis, treatment, and prognosis during follow-up of all patients are summarized retrospectively. *Results*. Four cases were trended to be AMH and 6 cases were trended to be pheochromocytoma before operation; moreover, the other two patients were diagnosed accidentally. All patients, except for the patient with mucinous tubular and spindle cell carcinoma of left kidney by open surgery, experienced a smooth laparoscopic adrenalectomy, including 2 with radical nephrectomy, 10 of which experienced unilateral adrenalectomy, 1 was bilaterally partial adrenalectomy, and the remaining one was unilaterally complete removal and then 2/3 partially contralateral excision. After a medium follow-up of 6.5 years, it demonstrated a satisfactory outcome of 8 cured patients and 4 symptomatic improved patients. *Conclusions*. AMH presents a mimicking morphology and clinical manifestation with pheochromocytoma. Surgery could be the only effective choice for the treatment of AMH and showed a preferable prognosis after a quite long follow-up.

## 1. Introduction

Catecholamine syndrome is a frequent factor leading to hypertension, including adrenal medullary hyperplasia (AMH) and pheochromocytoma. However, AMH is much rarer than pheochromocytoma, which is characterized by paroxysmal hypertension clinically and adrenal medullary cell mass hyperplasia pathologically [1]. Patients with AMH are frequently misdiagnosed to have pheochromocytoma as similar manifestations, laboratorial results, and radiological presentations.

We aim to analyze the detailed characteristics, share the experience of surgical treatment and prognosis of AMH, and provide some possible advices of distinguishing AMH from pheochromocytoma through a systematic review of literatures and cases in hand.

## 2. Materials and Methods

Twelve patients were pathologically diagnosed as AMH from January 1999 to December 2013 in our hospital. All data were searched and collected retrospectively.

Before operation, imaging examinations, including urinary color Doppler ultrasonography (US), enhanced computed tomography (CT), magnetic resonance imaging (MRI), and ^131^I-metaiodobenzylguanidine (^131^I-MIBG) scintigraphy, were used to confirm and identify the position and details of adrenal lesions. When specific or suspicious lesions were ascertained, adrenal hormones were routinely detected, including serous electrolytes, cortisol, aldosterone, and 24-hour catecholamine in blood and 17-hydroxy (17-OH), 17-ketones (17-KS), vanillylmandelic acid (VMA), and 24-hour catecholamine in urine. In addition, Regitine suppression test was conducted.

Perioperative prescriptions, including one to four weeks of routinely oral *α*-adrenergic receptor blockers, phenoxybenzamine (usually from 5 mg b.i.d to maximal 30 mg t.i.d) or prazosin (usually from 1 mg q.d. to maximal 3 mg t.i.d), and 3 days of alternately transvenous use of a dose of 1000 mL crystalloid and colloidal solutions, respectively, were applied to reduce the incidence of turbulence of haemodynamics during operation. For patients with tachycardia, different doses of *β*2-receptor blockers, such as Betaloc, would be used till heart rate was less than 90 per minute.

After all preparations were completed, laparoscopic surgery through retroperitoneal approach would be carried out on all patients. According to results of imageology, unilateral adrenal gland would be abscised in patients with significantly one-side hyperplasia or masses, while bilaterally subtotal adrenalectomy could be taken into consideration when bilateral masses were found. Severer side could be removed in patients with bilateral hyperplasia, and if clinical manifestations could not be controlled after surgery, contralateral partial adrenalectomy would be considered. Blood pressure, laboratory studies (mainly catecholamine), and imageology examinations were routinely conducted during follow-up.

## 3. Results

In summary, 10 patients complained of hypertension, 1 of paroxysmal blood urine, and 1 of renal mass merging elevating blood pressure during health examination. In patients with only hypertension, 6 were persistent and 4 were paroxysmal. The patients complaining of blood urine and renal mass were diagnosed to have right nephrectomy combined with nephrolith and mucinous tubular and spindle cell carcinoma (MTSCC) of left kidney, respectively. Unilateral nephrectomy was carried out in these two patients, 1 by laparoscopic surgery and the other one by open surgery, and then AMH was accidentally diagnosed, which made all examinations focus on adrenals neglected. Therefore, all examinations mentioned were excluded for these two cases. All patients had a degree of slight to occasionally severe headache when ascending of blood pressure seizure, with a possible inducement of tension, overworked or position altering, and so forth. Detailed characteristics of these patients were summarized in Table 1.

All patients had no evident abnormalities in serous electrolytes (mainly potassium), cortisol, and aldosterone, whereas different degrees of elevating could be demonstrated in uric 17-OH, 17-KS, vanillylmandelic acid (VMA), and uric and serous catecholamine, respectively. Regitine suppressing tests were all positive.

Enlargement of unilateral adrenal and low-echoes of adrenal masses were shown in 4 cases and 3 cases, respectively, based on urinary US, and no bilateral lesions were found. According to CT scans, 4 cases of bilateral abnormalities and 6 cases of unilateral abnormalities could be found. In patients with bilateral lesions, 1 patient was diagnosed to have bilateral masses, 2 to have unilateral mass and contralateral hyperplasia, and 1 to have bilateral hyperplasia. However, in patients with unilateral lesion, both hyperplasia and mass were diagnosed in 3 cases, respectively. The sizes of masses ranged from 0.3 to 1.2 cm, except that one adrenal mass was sized of about 3.0 cm but diagnosed as adrenocortical adenoma. For the patient with MTSCC, 1.5 cm of mass in his left adrenal was reported.

Further, 6 patients with adrenal hyperplasia had experienced a MRI scan, which showed nearly similar results with CT scan, except for 1 case of no anomaly in MRI. ^131^I-MIBG was applied in 6 patients with hyperplasia and showed enhanced signal gathering. No ectopic gatherings were found in ^131^I-MIBG.

In total, 4 cases were suspected to be AMH while 6 were suspected to be pheochromocytoma before operation. All patients underwent a smoothly, unilaterally, and retroperitoneally laparoscopic adrenalectomy, except for 1 with bilateral partial adrenalectomy and 2 with radical nephrectomy, without any evident hemodynamic instability during operation. Left AMH and right adrenocortical adenoma were then diagnosed in patient suffering from bilaterally partial surgery. After a routinely postoperative follow-up of 1 month, 1 patient with unilateral mass and contralateral hyperplasia complained of unsatisfactorily blood pressure (BP) controlling and received a reoperation of 2/3 left partially excision and 12 months of phasedown of hormone replacement therapy with nearly normal BP.

Pathology showed that (1) 14 adrenals from 12 patients were macroscopic hyperplasia and bodiness, of which mass could be found in 9 adrenals from 8 patients with a diameter ranging from 0.2 to 3.0 cm, (2) increased medulla could be found in all samples with a corticomedullary ratio ranging from 1 to 10, from which medulla could be found in all of adrenals, (3) consequently, 13 specimens excised were diagnosed to be AMH and four patients were diagnosed to have diffuse hyperplasia while other 8 were diagnosed to have nodal, with a diameter ranging from 0.2 to 1.5 cm, and multiple nodes were found in 1 patient, (4) cortical lesions had been merged in 3 patients, with 2 adrenocortical adenomas and 1 nonfunctional adenoma, and (5) MTSCC of kidney and ganglioneuroma were diagnosed in 1 patient, respectively.

After a medium follow-up of 6.5 (range: 1–14) years, 8 patients were cured with almost normal BP, symptoms, examinations, and studies and 4 patients obtained a significant improving of discomforts and decreasing of BP with only 2 patients who need a low dose of single antihypertensive drug. The patient receiving unilateral total and contralateral subtotal surgery acquired a compensatory hyperplasia of the remaining adrenal tissue and recovered to orthobiosis without of hypotensor taken.

## 4. Discussion 

AMH, usually and bilaterally, is an infrequently clinicomorphologic entity which is characterized by hypertension clinically and medullary hyperplasia pathologically. It has been reported to be associated with familial or type 2 multiple endocrine neoplasia (MEN 2) syndrome and is regarded as a precursor of pheochromocytoma [2].

The etiology of AMH remains unknown, which may be caused by a synthetic of heredity, nerve system, and endocrine system. Quantities of susceptibility genes mutation, including RET, SDHB, and NF-1, have been recognized as being possible to the development of AMH to pheochromocytoma [3–6]. Multiple limitations, such as deficient cases, insufficient basic researches, and difficulties of establishing models, have made the pathology, diagnosis, and therapy of AMH indefinite and controversial for a long time [7].

Although the primary diagnosis of AMH in our study mainly has relied on imageology, including US, CT, and MRI, the effectiveness of these technologies is still under debate. Yung et al. have presented the superior diagnostic sensitivity of MIBG scans through a case without abnormality in CT and MRI [8]. Similarly, the advantages of ^131^I-MIBG in the diagnosis of MEN 2 have been verified [9]. Combination application of MRI and MIBG could be more valuable for diagnosis of pheochromocytoma [10]. These conclusions might be similarly applied to AMH. Further, MIBG might provide a guidance of excluding ectopic lesions, which would be significant for protocols of treatment and prognosis. However, routine preoperative I-MIBG seems unnecessary for patients with pheochromocytoma [11, 12].

Additionally, after adrenal lesions were explicit or suspected, laboratory inspections of increasing of blood and urinary catecholamines could be helpful for distinguishing AMH from aldosteronism, cortical adenoma, and paragangliomas, but difficult from pheochromocytoma.

To prevent severe complications caused by AMH, surgery is still the most effective choice. Laparoscopic surgery has been prevalent for over a decade in our hospital, as quantities of advantages of no significant differences in security, smaller trauma, less blood loss and shorter hospital stay, and so forth, compared with the traditional open surgery. We would like to have an extraperitoneal approach which could exert less interference in gastrointestinal system. Similar outcomes and shorter hospital stay have been proven in retroperitoneoscopic adrenalectomy compared with traditional laparoscopic surgery [13–15]. In addition, the safeties and efficiencies of robot-assisted and laparoscopic single-site adrenalectomy compared to conventional laparoscopic surgery have demonstrated insignificant differences by systematic review and meta-analysis, respectively [16–18].

What is more, surgical techniques for adrenal neoplasms have experienced a variation from total adrenalectomy to unilateral adrenalectomy and to bilateral subtotal adrenalectomy with preservation of normal tissue [19]. Moreover, prophylactic bilateral adrenalectomy has not been advocated, as Addisonian crisis could occur in one-quarter of the patients over 10 years despite adequate corticosteroid replacement [20].

From our experience, patients with AMH should undergo a similar preoperative preparation with pheochromocytoma; then an adrenalectomy (partial or total) could be carried out at the evident side. If the symptoms could not be relieved, contralateral partial adrenalectomy should be taken into consideration. Importantly, bilaterally partial adrenalectomy should not to be neglected for patients with bilateral nodes. Bilateral adrenalectomy should be the final choice to avoid lifelong hormone replacements and complications of cortisol taking in. In our study, one patient received bilaterally subtotal adrenalectomy simultaneously, while another received 2/3 of left adrenalectomy at his one-month follow-up after right adrenalectomy. Both gained a quite satisfactory prognosis except one with 12 months of phasedown of replacement therapy.

Though macroscopic differences between pheochromocytoma and AMH seem inconspicuous, slight distinction could be detected. Valdés et al. reviewed literatures and tried to summarize the main differences of clinical and pathological features between pheochromocytoma and AMH [21]. However, small lesions could be hard to be distinguished macroscopically according to this principle. Additionally, some believed that pheochromocytoma, mostly unilateral, is a hypostatic neoplasm which has integral capsule, while adrenal medulla out of neoplasm would be normal or at least not be atrophic though been extruded; however, AMH usually to be bilateral with diffuse or small nodular alterations in medulla as well as contrast presentations in capsule and invasion of entity compared with pheochromocytoma.

Above all, pathology is still the only method to confirm AMH. Pitifully, uniform standards concentrating on pathological diagnosis are lacking. In our opinion, the main criteria about pathological diagnosis of AMH are decreasing corticomedullary ratio with a quotient less than 10 combined with elevating weight of adrenal medullary; medullary hyperplasia is a proliferation of cells containing normal cellular architecture as opposed to the nests of cytologically atypical polygonal cells which characterize pheochromocytoma [22]; medullary could be found in alar part or tail of adrenal with or without nodular hyperplasia and polymorphic cells could exist; furthermore, medullary hyperplasia with small nodes without capsule or only medullary hyperplasia without neoplasm could be also one reference. However, pheochromocytoma but AMH should be diagnosed, no matter the size of entity when capsule or tumor cells larger than normal pheochromocytes microscopically appear. Meanwhile, medullary that appears in tail of adrenal could not be the evidence of AMH. Equally, AMH could not be excluded without medullary in tail.

Our study trended that AMH would be a benign lesion with mild biological behavior. Similar conclusion was presented by Lenders et al. [23]. However, there is still 30%–50% risk to develop contralateral tumors after unilateral adrenalectomy in MEN 2 within 10 years [20]. According to our study, all patients gained preferable efficacy in a mean follow-up of 6.5 years, with no recurrence or malignant transformation.

## 5. Conclusion

AMH is a rare disease which presents a mimicking morphology and clinical manifestation with pheochromocytoma. Combination of medical history, imageology, and laboratory tests could only provide a tendency in its diagnosis, though MIBG might have a superior advantage. Though surgery could be the only effective choice, surgical modes should be evaluated thoroughly. Preferable prognosis after a quite long follow-up was present.

## Figures and Tables

**Table 1:** Clinical characteristics of patients.

	Male	Female	Total
	number of patients	number of patients	
Total	8	4	12
Medium age (range)	47 (39–71)	33.5 (27–45)	41.5 (27–71)
Symptoms			
Hypertension	7	3	10
Headache	3	2	5
Dizziness	4	2	6
Nausea and vomiting	3	1	4
Heart anomalies	2	1	3
Operation			
Laparoscopic	7	4	11
Open	1	—	1
Lesions			
Hyperplasia only	1	3	4
Mass only	4	1	5
Hyperplasia and mass	3	0	3
Size of mass (cm)			
Medium	0.5	0.5	0.5
Range	0.2–1.5	—	0.2–1.5
Follow-up (yr)			
Medium	6.5	4.5	6.5
Range	2–14	1–9	1–14
